# Stakeholder perspectives on generative AI in education: A dataset of Reddit posts and comments (2022–2025)

**DOI:** 10.1016/j.dib.2026.112871

**Published:** 2026-05-22

**Authors:** Prabha M. Kumarage, Sachini Gunasekara, Mirka Saarela

**Affiliations:** Faculty of Information Technology, University of Jyväskylä, P.O. Box 35, FI-40014 Jyväskylä, Finland

**Keywords:** Generative AI, Reddit, AI in education, Public discourse, Sentiment analysis

## Abstract

This data article describes a dataset capturing public discourse on generative AI (GenAI) in education, collected from Reddit between 1 September 2022 and 31 October 2025. The dataset comprises 984 unique posts and 8346 associated comments drawn from 320 education-related subreddits, grouped into three stakeholder categories: students, educators, and parents. The posts and comments focus on how GenAI tools, such as ChatGPT, are used, experienced, and debated in relation to teaching, learning, assessment, and broader educational practices. Data were retrieved via the Reddit API and processed through a privacy-preserving pipeline that removed personal identifiers in accordance with platform policies. Each entry is accompanied by rich metadata, including a unique identifier, content type (post or comment), stakeholder group, subreddit, creation timestamp, and engagement metrics. Spanning the period before and after the introduction of ChatGPT, the dataset enables temporal analyses of discourse volume and content evolution. This openly available dataset provides a valuable resource for research in education, human–AI interaction, social media analytics, and natural language processing, and supports reproducibility in studies of public engagement with GenAI technologies.

Specifications Table

**Table:** 

Subject	Computer Sciences
Specific subject area	Public discourse on GenAI in education; stakeholder-specific analysis of Reddit posts and comments discussing AI and learning.
Type of data	Data 1: Unfiltered and Unlabeled; Data 2: Filtered and Labeled; Table.
Data collection	Data were collected from Reddit using the Python Reddit API Wrapper (PRAW) and the official Reddit API. The collection covered the period between 1 September 2022 to 31 October 2025 and targeted 320 education-related subreddits identified through keyword filtering and manual validation. Posts and comments referencing GenAI tools were retrieved and stored together with their associated metadata. All personal identifiers were removed during preprocessing, and stakeholder categories (students, educators, and parents) were assigned based on the corresponding subreddit type.
Data source location	Reddit.
Data accessibility	Repository name: Mendeley Data Data identification number: 10.17632/z6794ft3fs.3 Direct URL to data: https://data.mendeley.com/datasets/z6794ft3fs/3 A README file included in the repository describes the file structure, explains how each dataset file corresponds to the tables reported in this article (Table 1 corresponds to Data 1 and Data 2, Table 2 corresponds to Data 2), and provides detailed documentation of metadata, data cleaning procedures, stakeholder labeling criteria, and dataset versioning information. In addition, the repository provides a sanitized workflow code (reddit_genai_dataset_workflow.ipynb) illustrating the data collection and preprocessing steps. The repository also includes a supplementary CSV file (Subreddits.csv) listing all education-related subreddits used for data collection, together with their stakeholder categorization.
Related research article	none.

## 1 Value of the Data

• This dataset provides a comprehensive and domain-specific view of discussions on generative artificial intelligence (GenAI) in education on the major social platform Reddit, enabling analyses of how key stakeholder groups–students, educators, and parents–discuss, evaluate, and adapt to GenAI within educational contexts. Unlike general social media datasets, it includes explicit stakeholder labeling and focuses specifically on GenAI in educational settings. Analyses can draw on the stakeholder_group, specific_subreddit, and textual fields (title, text), for example, to compare how educators and students discuss academic integrity or classroom use of GenAI tools. As the data are platform-specific, the insights reflect Reddit discourse and should not be interpreted as representative of the broader educational population.
• The dataset spans periods before and after the public release of ChatGPT, supporting temporal analyses of discussion volume, topical focus, and patterns of stakeholder engagement over time using the created_utc timestamp in combination with post counts or content features. For instance, researchers can examine how discussions on AI-assisted assignments or cheating change over time or compare pre- and post-ChatGPT discourse trends.
• The dataset supports exploratory and descriptive analyses of education-relevant themes such as stakeholder discussions of academic integrity, learning support, assessment practices, and policy-related issues associated with GenAI use in education. For example, researchers can investigate how discourse around plagiarism detection evolves or identify differences in dominant topics across students, educators, and parents. These analyses are supported by the stakeholder-labeled, longitudinal structure as well as the availability of both unfiltered and manually validated versions of the dataset.
• The dataset can be reused to train and evaluate sentiment analysis, emotion classification, and topic modeling approaches focused on educational technology and AI-related discourse on social media. For instance, models can be trained on the text field and evaluated across stakeholder_group categories to examine differences in sentiment or emotional expression.
• Researchers can also use the dataset for comparative studies across stakeholder groups, longitudinal analyses of GenAI adoption narratives, and as a benchmark dataset for explainable natural language processing and social media analytics in educational research. For example, stakeholder_group can be used as the target label in a three-class perspective classification task, where models predict whether an entry reflects a student, educator, or parent perspective using the textual fields, and performance can be evaluated using macro-F1 to account for class imbalance. However, the parent group is very small in the filtered dataset (69 entries; 0.7% of all entries), and analyses involving parents should therefore be treated as exploratory. This imbalance likely also reflects lower levels of parent participation in Reddit discussions on GenAI in education relative to students and educators.
• The dataset is openly available and anonymized, supporting reproducibility and ethical reuse in compliance with platform terms of service. All metadata fields and data preprocessing steps are transparently documented in the accompanying repository README, distinguishing the dataset from less structured social media collections and facilitating reproducible workflows.

## 2 Background

Education is undergoing a profound transformation driven by the rapid rise of GenAI tools such as ChatGPT, Gemini, and Claude. These large language models (LLMs) present both opportunities and challenges for students, educators, and parents, reshaping teaching, learning, and engagement with technology [[1], [2], [3], [4]]. Students often report that GenAI facilitates learning, provides feedback, and supports creative problem-solving, while also expressing concerns about overreliance and reduced independence [5,6]. Similar ambivalence appears in analyses of student discourse on Reddit, where enthusiasm coexists with anxiety about GenAI’s implications for education [[7], [8], [9], [10]].

Educators acknowledge GenAI’s potential to personalize instruction and reduce administrative workload but remain concerned about academic integrity and fairness [[11], [12], [13], [14]]. Online discussions also highlight worries about job security, institutional expectations, and ethical boundaries [7,15,16]. Parental perspectives are less documented; existing studies suggest many parents are unaware of their children’s GenAI use yet recognize both benefits and risks, with attitudes shaped by personal familiarity [17,18].

Most prior studies rely on surveys and interviews, and less is known about how stakeholder groups discuss GenAI organically in online spaces. Reddit provides a rich source of naturally occurring discourse. The dataset described in this article compiles such content from September 2022 to October 2025, covering both the pre- and post-ChatGPT phases, with posts organized by stakeholder group.

## 3 Data Description

The dataset contains Reddit posts and comments related to GenAI in education, collected between 1 September 2022 and 31 October 2025. Data retrieval was conducted using the Python Reddit API Wrapper (PRAW) and the official Reddit API, resulting in an initial corpus of 49,003 rows. This unfiltered dataset, released as Data 1 (Unfiltered and Unlabeled), includes all posts and comments that matched at least one GenAI-related keyword during retrieval. The abstract figure of 984 unique posts and 8346 associated comments refers specifically to Data 2 (Filtered and Labeled), which contains 9330 manually validated entries after deduplication and filtering.

Each row corresponds to either a top-level post or an individual comment and contains the variables as described in Table 1. A complete data dictionary is included in the repository README file, documenting variable names, definitions, data types, permissible values, and example entries to support secondary reuse. These variables provide a consistent structure across both posts and comments to support downstream natural language processing and qualitative analysis. Data 1 corresponds to the full corpus described in Table 1, which summarizes all variables for posts and comments prior to filtering.

**Table 1:** Open-sourced data details for Data 1.

Variable	Data Type	Description
**id**	String	Unique identifier of the Reddit post or comment automatically assigned during data extraction.
**post_id**	String	Unique identifier of the parent post to which a comment belongs; identical to id of top-level posts.
**type**	Categorical	Specifies whether the entry is a post or a comment.
**specific_subreddit**	String	The exact subreddit where the item was posted.
**title**	String	Title of the top-level post.
**text**	String	Text body of the post or comment.
**created_utc**	Date-time	UTC timestamp of creation.
**score**	Integer	Upvote score at the time of retrieval.
**num_comments**	Integer	Total number of comments on the post.

Data 1 therefore represents the primary intermediate output of the pipeline prior to deduplication and manual filtering. It enables users to inspect the raw keyword-based retrieval results and assess how subsequent cleaning and validation steps affected dataset composition. Detailed counts are reported for each processing stage below, and the repository README further documents the transition between dataset versions.

A second version of the dataset, Data 2 (Filtered and Labeled), contains a filtered and manually validated subset of Data 1. This version includes only posts and comments that explicitly reference both GenAI and an educational context. Because the collection process involved iterative API calls over multiple time windows, 7659 duplicate rows were detected and removed from Data 1, resulting in 41,344 unique entries after deduplication. After automated filtering and manual screening to exclude irrelevant use cases, 9330 rows remained in Data 2, comprising 984 unique posts and 8346 associated comments.

Data 2 also includes an additional variable, shown in Table 2, which assigns each row to one of three stakeholder groups: students, educators, or parents, based on subreddit categorization and manual verification. Data 2 builds on the structure of Data 1 and is described in Table 2, which documents the additional stakeholder_group variable included in the filtered dataset.

**Table 2:** New field introduced in Data 2.

Variable	Data Type	Description
stakeholder_group	String	Stakeholder group assigned to each row: students, educators, or parents.

Table 1, Table 2 together provide a complete overview of all variables included in the dataset, including their definitions and data types, for both the Data 1 and Data 2 versions. Example records for both posts and comments are included in the released spreadsheet files, allowing users to directly inspect the data structure. Together, Data 1 and Data 2 provide a structured and openly accessible resource for examining public discussions of GenAI in education. Both datasets are released in Microsoft Excel (.xlsx) and Comma-Separated Values (.csv) formats. While Data 1 and Data 2 contain the same core variables, the column order in Data 2 has been reorganized to improve logical structure and readability; the variable order presented in Table 1 reflects the structure of Data 2. In the dataset, all personal identifiers were removed in accordance with Reddit's terms of service.

The dataset is released as version 3.0 on Mendeley Data, and any future updates will be provided as versioned releases with persistent DOIs to ensure reproducibility.

The filtered dataset (Data 2) contains 9330 entries distributed across stakeholder groups, including 6514 entries from educators (69.8%), 2747 from students (29.4%), and 69 from parents (0.7%), indicating an uneven distribution of participation across groups. This low parent representation may partly reflect that parental concerns about GenAI in education were more visible in discussions about younger or school-aged children than older high-school, college, or university students. The dataset spans the period from September 2022 to October 2025. No relevant entries were identified prior to November 2022, and activity increases over time, with higher volumes observed in later periods of the dataset. A total of 320 education-related subreddits were included, comprising 164 student-oriented, 95 educator-oriented, 24 parent-oriented, and 37 general education subreddits, as detailed in the supplementary file (Subreddits.csv). These descriptive statistics provide an overview of dataset composition and highlight potential imbalances across stakeholder groups, time periods, and subreddit activity.

## 4 Experimental Design, Materials and Methods

The dataset was generated through a multi-stage pipeline designed to produce a clean, domain-specific corpus focused on public discourse about GenAI in education.

The overall data collection and preparation workflow was guided by principles from the CRISP-DM data mining framework, encompassing phases of data understanding, data acquisition, data preparation (including cleaning and filtering), and data documentation for reuse [19]. While the present work does not follow a formal implementation of a single framework, these principles informed the design of the pipeline described below.

**Fig. 1:**
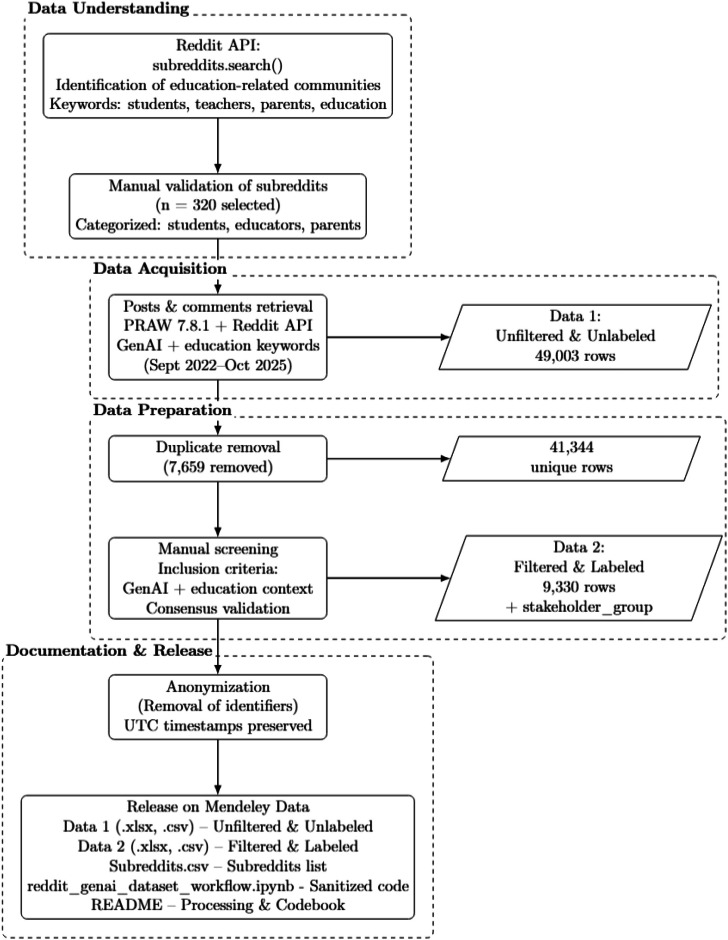
Overview of the Reddit data collection and reduction pipeline.

Specifically, the data collection and preparation process followed a structured, multi-step workflow: (1) identification and manual validation of education-related subreddits; (2) retrieval of posts and comments via the Reddit API using predefined GenAI-related keywords; (3) removal of duplicate entries resulting from iterative API queries; (4) manual screening to retain only content referencing both GenAI technologies and educational contexts; (5) stakeholder group assignment based on subreddit categorization and content verification; and (6) anonymization prior to data release. Fig. 1 summarizes this data collection, reduction, and release pipeline. In summary, the initial keyword-based retrieval yielded an unfiltered corpus (49,003 entries), which was subsequently deduplicated and manually screened to produce a filtered and stakeholder-labeled dataset (9330 entries), as described step-by-step in the workflow above.

Candidate subreddits were identified using the Reddit API by querying for relevant communities via the subreddits.search() function, using the keywords students, teachers, parents, and education. This process returned 803 initial subreddit search results across the four seed keywords, including some duplicate or redundant subreddit entries returned across multiple keyword searches. All API-returned candidate results were manually reviewed based on subreddit titles, descriptions, and community purpose. After removing redundant entries and excluding communities that were not relevant to educational contexts, 320 education-related subreddits were selected for data collection. Because this is a social media dataset, no participants were recruited directly; instead, all posts and comments were drawn from publicly accessible education-related subreddits.

All retrieved communities were then manually examined based on their titles, descriptions, and community purpose. Subreddits were selected based on their relevance to educational contexts, as determined from these characteristics. Through this manual review process, a total of 320 subreddits were selected and categorized into three stakeholder groups: students, educators, and parents. Because the full list of subreddits is extensive, it is provided as a separate supplementary file in the Mendeley Data repository associated with this article (Subreddits.csv).

All posts and comments originating from a given subreddit initially inherited that subreddit's stakeholder label as the stakeholder_group variable in Data 2. In rare cases where the content or author clearly indicated that the contributor belonged to a different stakeholder group (e.g., a parent posting within a student-oriented subreddit or an educator commenting in a student forum providing teaching advice), manual reassignment was performed to ensure accurate stakeholder labeling, as documented in the repository README to support transparency and replicability.

Data extraction was based on a comprehensive keyword list targeting GenAI-related terminology and AI-in-education contexts. The keyword list was designed to balance recall and relevance by combining GenAI-specific terms with education-related usage contexts. The list included the following keyword groups used during API queries:

Primary GenAI Keywords:

chatgpt, openai, gpt, gpt-4, gpt4, bard, claude, gemini, microsoft copilot, copilot, llm, large language model, generative ai, genai

Education-specific GenAI Keywords:

ai in education, ai in classroom, ai cheating, ai essay, ai writing tools, ai tutor, ai grading, ai feedback, ai exam, ai-written essay, ai assignments, ai academic integrity, plagiarism with ai, turnitin ai, school using ai, student using ai, teacher using ai, ai learning, ai misuse, academic dishonesty ai

These keywords were applied during initial Reddit API retrieval to capture posts and comments related to GenAI and reduce retrieval of unrelated content. The same keyword list was applied consistently across the entire data collection period (September 2022–October 2025) to ensure comparability over time. For education-specific keywords, however, we also included the more general term AI. This choice was intentional because the general public frequently refers to GenAI simply as AI, and many posts discussing ChatGPT or other LLMs do not explicitly use the term GenAI [20]. However, this broader strategy also resulted in the retrieval of posts that used AI in non-GenAI contexts. Those non-GenAI items were later identified and removed during manual cleaning to ensure that Data 2 contains only discourse specifically related to GenAI in education settings.

Reddit posts and comments were collected using the Python Reddit API Wrapper (PRAW – version 7.8.1) and the official Reddit API. Entries were retrieved if they matched at least one keyword and originated from one of the selected subreddits, and all 49,003 entries are available on Data 1 (Unfiltered and Unlabeled). Each row includes metadata such as creation timestamp, score, subreddit name, title (for posts), and text content. Engagement-related variables (e.g., score and number of comments) reflect a single snapshot at the time of data retrieval and were not tracked longitudinally or versioned across collection dates. All timestamps are stored in UTC to support temporal and cross-platform analyses. Preprocessing steps included the removal of usernames and other personal identifiers.

The data collection protocol is summarized as follows: Queries were executed in batches of up to 10 subreddits at a time rather than via a single global Reddit search in order to ensure stakeholder-specific coverage while complying with API constraints. Within each query batch, GenAI-related and education-related keyword lists were applied using OR logic, and posts were retained during initial retrieval if they matched at least one keyword in the post title or body text. All comments associated with retrieved posts were subsequently collected through follow-up API calls. To cover the period from September 2022 to October 2025 and to avoid API result limits, data collection was conducted in sequential temporal windows of three or four months. Pagination was handled through the Reddit API listing mechanisms until no further results were returned for each subreddit batch and time window. Reddit API rate limits were respected by incorporating automated pauses between requests and retry logic for transient failures. Post and comment identifiers were stored across collection rounds to allow detection and removal of duplicate entries resulting from overlapping keyword queries or adjacent time windows. The complete keyword lists, dataset structure, cleaning procedures, stakeholder labeling rules, and versioning information are further documented in the accompanying repository README to support independent replication. This structured query design ensures reproducibility of the data collection process.

The following pseudocode summarizes the operational steps used to generate Data 1 and Data 2:

**Pseudocode:** Reddit Data Collection and Filtering Pipeline.

**Table:** 

**Require:** Reddit API access, search keywords, GenAI_terms, Education_terms **Ensure:** Data 2 (Filtered & Labeled Dataset) **1. Subreddit Identification and Validation** 1. Search Reddit communities using subreddits.search() **Keywords** ← {students, teachers, parents, education} 2. Manually validate selected subreddits 3. Assign stakeholder_category ← {students | educators | parents} 4. Save Validated_Subreddit_List **2. Initialization** 5. Load Validated_Subreddit_List 6. Define GenAI_Terms 7. Define Education_Terms **3. Data Collection** 8. **for each** Time_Window (3–4 months) **do** 9. **for each** Subreddit_Batch (≤10 subreddits) **do** 10. Query Reddit API using OR-combined (GenAI_Terms ∪ Education_Terms) 11. Retrieve posts matching ≥1 term in title or body text 12. Retrieve all comments for matching posts 13. Append records to Corpus 14. Store Post_IDs and Comment_Is 15. **end for** 16. **end for** 17. Save Corpus as Data_1 (Unfiltered & Unlabeled) **4. Deduplication** 18. Deduplicate Data_1 using Post_IDs and Comment_IDs **5. Filtering and Labeling** 19. **for each** Record in Data_1 **do** 20. **if** Record refers to GenAI **AND** education context **then** 21. Retain Record 22. **else** 23. Exclude Record 24. **end if** 25. **end for** 26. Assign Stakeholder_Group from Subreddit 27. Manually reassign rare ambiguous cases by consensus 28. Save as Data_2 (Filtered & Labeled) **6. Anonymization and Export** 29. Remove usernames and personal identifiers 30. Preserve UTC timestamps 31. Export CSV/XLSX files and documentation

Because data were collected iteratively through repeated API calls over multiple dates, the dataset contained duplicate entries; 7659 duplicates were removed, resulting in 41,344 unique rows. Although keyword filtering removed much irrelevant content, posts sometimes contained GenAI-related keywords while discussing unrelated domains. To address this, every row in Data 1 was manually screened.

Manual validation was conducted by two authors using a shared set of predefined inclusion and exclusion criteria. Specifically, each post or comment was retained only if it (i) explicitly referred to a GenAI technology and (ii) was situated within an educational context, such as teaching, learning, assessment, or educational policy. Content was excluded if it referred to AI in non-generative contexts or discussed GenAI without a clear educational connection. In total, manual screening reduced the post-deduplication corpus from 41,344 unique rows to 9330 retained rows, meaning that 32,014 rows were excluded because they did not meet the combined GenAI-and-education inclusion criteria. These exclusions included non-GenAI uses of AI, entries without a clear educational connection, and other records outside the dataset scope. These criteria were documented in a brief annotation guideline to ensure consistent application across the dataset and are further described in the accompanying repository README.

The validation and stakeholder labeling process followed a consensus-based approach. Each item was reviewed by at least one author, and ambiguous cases were jointly discussed by two authors, with input from a third author where necessary, until consensus was reached. Based on the authors’ validation notes and recollection, fewer than 20 entries required joint discussion or third-author input, corresponding to <0.05% of the 41,344 manually screened unique records. Stakeholder labels were primarily assigned based on subreddit categorization and manually adjusted in cases where the content clearly indicated a different stakeholder perspective. Because the final labels were derived through consensus-based rather than independent parallel annotation, no formal inter-annotator agreement statistics were calculated. This approach prioritized consistency and domain relevance while ensuring transparency through detailed documentation of the annotation procedure.

After validation, 9330 rows remained and were included in Data 2 (Filtered and Labeled). This version adds the stakeholder_group variable, indicating the stakeholder group for each row. Aside from duplicate removal and manual exclusion of irrelevant items, no additional statistical outlier filtering was applied; missing values reflect unavailable metadata returned by the Reddit API (e.g., empty titles for comments).

The time coverage of the dataset allows examination of both the early, low-volume pre-ChatGPT period (before November 2022) and the rapid expansion of discourse that followed the public release of ChatGPT and later GenAI models such as Claude, Gemini, and Microsoft Copilot. All timestamps are provided in UTC to support temporal or cross-platform comparisons. Both Data 1 and Data 2 are available in spreadsheet (.xlsx) and comma-separated values (.csv) formats and are hosted on Mendeley Data, with all personal identifiers removed in accordance with Reddit’s terms of service. The dataset preserves the full textual content of posts and comments along with essential metadata, enabling analyses of discourse patterns, sentiment, stakeholder perspectives, topic evolution, and other computational social science applications related to GenAI in education. While no analytical comparisons are performed in this data article, the dataset is explicitly structured to support comparative analyses across stakeholder groups, dataset versions (unfiltered vs. filtered), and time periods (pre- and post-ChatGPT release).

## Limitations

The dataset includes only publicly available Reddit discussions from selected education-related subreddits and may not represent all stakeholder perspectives or the full diversity of educational discourse across platforms or populations. Keyword-based retrieval may have excluded relevant posts without the specified terms and included some only loosely related to GenAI or education.

Although stakeholder categorization was conducted through subreddit grouping and manual checks, some misclassification may remain. Manual validation and stakeholder labeling relied on consensus-based decisions rather than independent parallel annotation with formal inter-annotator agreement statistics, so some subjectivity may persist. Engagement metrics reflect a single snapshot at the time of collection and may change thereafter; longitudinal updates are not included. Intermediate processing logs and snapshots generated during collection and cleaning are not publicly released, which may limit full auditability. This is partly mitigated through sanitized workflow code and accompanying documentation. As discourse evolves rapidly alongside GenAI technologies, earlier and later posts may differ in vocabulary and context, affecting temporal comparisons.

For secondary analyses, users should address class imbalance across stakeholder groups or time periods, and temporal sparsity before ChatGPT’s public release. Using time-window aggregation or resampling strategies may support responsible and effective reuse of the dataset.

## Ethics Statement

The authors have read and followed the ethical requirements for publication in Data in Brief. The dataset described in this article consists exclusively of publicly available social media content collected in compliance with the Terms of Service of Reddit. No direct interaction with users occurred, and no private or non-public data were accessed. All textual data have been anonymized to remove usernames, URLs, or any other identifying information. Consequently, the dataset does not constitute human subjects research as defined by institutional review boards, and no ethical approval was required.

## CRediT Author Statement

**Prabha Kumarage:** Conceptualization, Methodology, Software, Programming, Data Preprocessing, Writing- Original draft preparation, Writing- Reviewing and Editing. **Sachini Gunasekara:** Conceptualization, Data Preprocessing, Writing- Original draft preparation. **Mirka Saarela:** Conceptualization, Methodology, Writing- Original draft preparation, Writing- Reviewing and Editing.

## Data Availability

Mendeley DataReddit discussions on generative AI in education dataset (Original data)

## Appendix Supplementary materials

Image, application 1
